# *Giardia duodenalis*: Number and Fluorescence Reduction Caused by the Advanced Oxidation Process (H_2_O_2_/UV)

**DOI:** 10.1155/2014/525719

**Published:** 2014-12-04

**Authors:** José Roberto Guimarães, Regina Maura Bueno Franco, Regiane Aparecida Guadagnini, Luciana Urbano dos Santos

**Affiliations:** ^1^Department of Sanitation and the Environment, School of Civil Engineering, Architecture and Urbanism, University of Campinas (UNICAMP), CP 6021, 13083-852 Campinas, SP, Brazil; ^2^Department of Animal Biology, Biology Institute, University of Campinas (UNICAMP), CP 6109, 13083-970 Campinas, SP, Brazil; ^3^Padre Anchieta University Center, UNIANCHIETA, Bom Jesus de Pirapora Street 100/140, 13207-270 Jundiaí, SP, Brazil

## Abstract

This study evaluated the effect of peroxidation assisted by ultraviolet radiation (H_2_O_2_/UV), which is an advanced oxidation process (AOP), on *Giardia duodenalis* cysts. The cysts were inoculated in synthetic and surface water using a concentration of 12 g H_2_O_2_ L^−1^ and a UV dose (*λ* = 254 nm) of 5,480 mJcm^−2^. The aqueous solutions were concentrated using membrane filtration, and the organisms were observed using a direct immunofluorescence assay (IFA). The AOP was effective in reducing the number of *G. duodenalis* cysts in synthetic and surface water and was most effective in reducing the fluorescence of the cyst walls that were present in the surface water. The AOP showed a higher deleterious potential for *G. duodenalis* cysts than either peroxidation (H_2_O_2_) or photolysis (UV) processes alone.

## 1. Introduction

The constant environmental degradation of watersheds near densely populated areas has greatly altered the quality of public water sources. Although access to safe drinking water is a priority, providing safe drinking water is challenging due to the potential for severe contamination and the lack of access to sanitary infrastructure. One billion people lack access to improved water supplies and 2.6 billion people lack adequate sanitation [1]. Waterborne diseases that are caused by microorganisms such as viruses, bacteria, and protozoa have been documented throughout the world. Parasitic protozoan diseases that are transmitted through water have caused 62.5 million disability adjusted life years (DALYs) worldwide.* Giardia duodenalis* (also known as* Giardia lamblia*) is parasite responsible for approximately 35% of gastrointestinal disease outbreaks caused by waterborne protozoa in Australia, Europe, and North America [2]. The economic losses, both direct and indirect, caused by* G. duodenalis* infection are considerable. Children are most at risk for the clinical consequences of* G. duodenalis* infection, particularly children in developing countries [3].

This protozoan is responsible for intestinal infection and diarrhea that may lead to nutritional deficiencies and significant morbidity and mortality, especially among children and the elderly. In areas where* G. duodenalis* is endemic, the emergence of resistant strains has led to a high rate of clinical failure of treatment [4].

Symptoms include diarrhea, malaise, flatulence, foul-smelling greasy stools, abdominal cramps, bloating, nausea, anorexia, and weight loss. Extraintestinal symptoms (urticaria, angioedema, and atopic dermatitis) have been reported, and a deficiency in the appropriate treatment of giardiasis has been identified [5]. Asymptomatic infection also occurs frequently (50–75% of infected persons). The absence of symptoms may result in a low clinical index of suspicion for diagnosis [6, 7].

*Giardia* spp. cysts (the environmentally resistant stage of the parasite) are transmitted by a fecal-oral route and are particularly suited to waterborne transmission because they are widely present in surface water, sewage, and drinking and recreational water [8]. Following ingestion, excystation takes place shortly after cysts leave the stomach and leads to rapid colonisation of the duodenum and jejunum, where the excysted trophozoites attach to the intestinal mucosa and multiply rapidly. Attachment is mediated by the ventral adhesive disc and is an essential feature of the relationship between* Giardia* spp. and its host and a prerequisite to sustained infection [9].

Infectious diseases remain a public health problem in Brazil. Although approximately 91.5% of the population has access to treated drinking water, the sewage generated from only 41.2% receives some treatment [10].* Giardia* spp. cysts were detected in many Brazilian environmental samples: wastewater, water (superficial and recreational), and bivalve mollusks [6, 11–14].

These cysts are highly prevalent in wastewater throughout various parts of the world, which may reflect the infection rate in the population. Although wastewater treatment plants are designed to produce effluents that carry low health-risk levels for the exposed population, treated wastewater can carry pathogens [6, 7].

The presence of* Giardia* spp. cysts can be reduced with wastewater treatment processes; however, it is difficult to achieve protozoa inactivation with the concentration of chlorine that is typically used for water and wastewater disinfection [7]. The disinfection step of treated sewage is crucial; however, most processes do not efficiently inactivate the resistant stages of the pathogens.

New procedures for pathogen disinfection, such as microwaves, high-intensity pulsed UV light radiation (PUV), and advanced oxidation processes (AOPs), have been evaluated (Guimarães et al., unpublished data) [15, 16].

The concept of the AOP, which originated in 1976 [17], entails processes that have the ability to generate elevated concentrations of hydroxyl radicals (^•^OH). Due to its high oxidative and nonselective character, (^•^OH) is a strong oxidant that is capable of complete oxidation of most organic compounds [18]. AOPs are also capable of promoting degradation of pharmaceutical compounds [19] and can inactivate bacteria such as* Escherichia coli* and the spore-forming bacteria* Clostridium perfringens* [20, 21].

The most direct method for generating hydroxyl radicals is the breaking of hydrogen peroxide by photolysis (H_2_O_2_/UV), as described in

(1) H2O2+hv⟶OH•+OH•

The destruction of microorganisms occurs as a function of oxidation of the cell wall and the subsequent cellular damage caused by the hydroxyl radical. This action leads to cell leakage and diffusion of the radical throughout the cell; the radical thus damages enzymes that affect protein synthesis [21, 22]. Few studies investigating the use of AOPs for the inactivation of protozoa in water have been reported [23, 24]. The use of UV photocatalysis/titanium dioxide (TiO_2_) for the inactivation of* Cryptosporidium* spp. oocysts has been evaluated [24].

The objective of the present study was to evaluate the efficiency of peroxidation associated with ultraviolet light (H_2_O_2_/UV) in reducing the number and fluorescence of* G. duodenalis* cysts.

## 2. Material and Methods

### 2.1. Experimental Method

**Figure 1:**
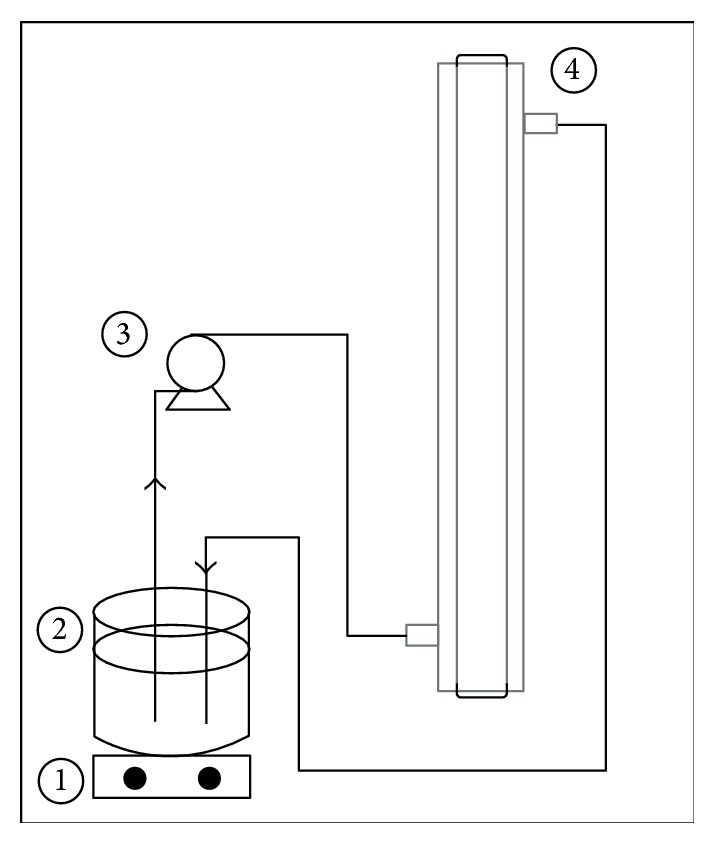
Experimental system: (1) magnetic stirrer, (2) reservoir (1 L), (3) peristaltic pump, and (4) reactor.

The experimental system consisted of a reactor designed by Guimarães and Barretto [20] (Figure 1) with a net volume of 190 mL (82 mL min^−1^). The photoreactors were operated in a one-way ascending passage mode for the three processes: advanced oxidation processing (AOP), peroxidation (H_2_O_2_), and photolysis (UV). The UV and peroxidation processes were used as a control to evaluate the synergistic effect of AOP (H_2_O_2_/UV).

All experiments were repeated six times for each process (*n* = 18), and the experimental system was decontaminated after each experiment with an elution solution of hypochlorite and ultrapure water (Milli-Q).

### 2.2. Samples Evaluated (Synthetic and Surface Water)

The aqueous solution (known as* synthetic water*) was formulated according to the proceedings of the American Public Health Association [25] using ultrapure water. Surface water was collected from the Atibaia River, which is the source of 95% of the water supply in Campinas (southeastern Brazil); these water samples were collected from the intake pipe in the Campinas water treatment plant of the municipal Society for Water Supply and Sanitation (SANASA). The plastic bottles that were used to obtain water samples were washed with 0.1% Tween 80 prior to collection to avoid the adherence of* Giardia* spp. cysts, which were naturally present in the water. In all the experiments 1 liter of raw water without artificial inoculation of* Giardia* spp. was processed. When the samples were positive, this number of cysts was subtracted for calculation of the reduction of organisms by processes.

The samples were transported to the laboratory under refrigeration at 10°C for same-day processing. Both water samples were seeded (under constant stirring) with 10^3^ *G. duodenalis* cysts that had been purified from fecal samples (A. L. Smith and H. V. Smith 1989 [26]). A hydrogen peroxide concentration of 12 g L^−1^ [27–29] and a UV radiation (*λ* = 254 nm) dosage of 5,480 mJ cm^−2^ were used in the experiments [30, 31]. Six experiments were performed for each of the three processes evaluated. We assessed two kinds of water, totaling 36 experiments.

### 2.3. Artificial Contamination of Samples with* Giardia* spp. Cysts

The cysts were taken from feces samples, according to A. L. Smith and H. V. Smith [26], and were diluted in PBS (1 : 9) in order to enumerate and prepare the inoculum. Enumeration was performed using RID, obtaining the number of cysts at 5 *μ*L (triplicate). The reading of these three aliquots was performed by 3 microscopists, without prior knowledge of the result of each count; the average number of* Giardia* spp. cysts was calculated for all conditions. Both samples of synthetic and superficial water were artificially contaminated with 10^3^ *Giardia* spp. cysts and submitted the processes studied (H_2_O_2_/UV, H_2_O_2_, and UV).

### 2.4. Sample Concentration

One liter of water samples (synthetic and surface) was filtrated by membrane cellulose ester membranes (Merck Millipore) with a diameter of 47 mm and a 3 *μ*m nominal porosity [32]. The filtrate was transferred to Petri dishes. The membranes were then scraped with a soft plastic loop and manually rinsed for 20 minutes with the eluting solution with Tween 80 (0.1%), which is a nonionic detergent that decreases the surface tension of water, allowing for the accommodation of nonpolar functional groups in the aqueous environment. It has been widely used as a component of eluting solutions to enhance protozoa recovery from filters surface. The resulting liquid was concentrated for 10 minutes (1,050 ×g), and the pellet was rinsed with ultrapure water for 10 minutes (1,050 ×g). Aliquots of 5 *μ*L of the two samples were dyed (fluorescein isothiocyanate, FITC), and the* G. duodenalis* cysts were enumerated using IFA staining followed by epifluorescence microscopy.

### 2.5. Molecular Identification

Molecular identification of* Giardia* in the samples was performed by polymerase chain reaction (PCR) of fragments of the Giardia *β*-giardin gene [33]. Characterization of the genotype of the* G. duodenalis* cysts that were present in the samples was performed through amplification and sequencing of a fragment of the glutamate dehydrogenate gene (GDH) [34].

### 2.6. Enumeration and Visualization of* G. duodenalis* Cysts Using an IFA

**Figure 2:**
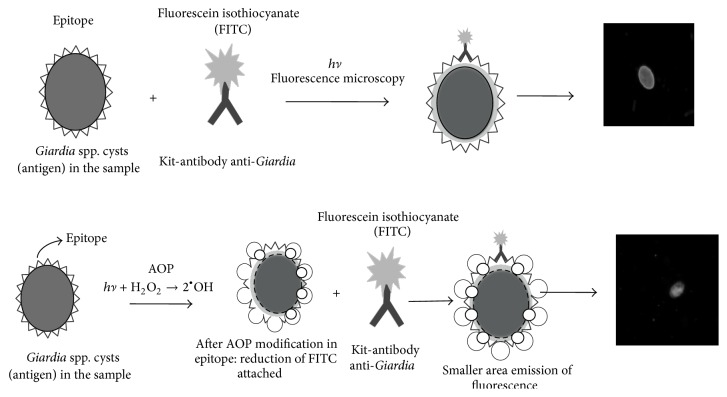
Scheme of the immunofluorescence assay (IFA): after AOP modification in epitope: reduction of anti-*Giardia* mAb-FITC conjugated binding.

The IFA is based on the reaction of anti-*Giardia* monoclonal antibodies with epitopes on the cyst walls. These antibodies are conjugated to a fluorochrome FITC used with a filter block (excitation 490 nm; emission 510 nm). The fluorochrome generates a predominant bright apple green color on the walls of the organism (Figure 2).

However, if the parasite cyst wall is damaged, antibody binding with the surface epitope will be hampered, and staining of the parasite will be reduced or eliminated. The lack of color suggests damage to the cyst wall surface. The IFA was performed with a Merifluor kit (Meridian Bioscience, Cincinnati, Ohio) according to the manufacturer's instructions, and the observational criteria described by Method 1623.1 of the United States Environmental Protection Agency [35] were used. The lower the recovery efficiency of* G. duodenalis* cysts, the higher the efficacy of the disinfection process.

### 2.7. Statistical Methods

The reduction means of the number of cysts were compared using Student's *t*-test and *F*-test both with *P* = 0.05.

## 3. Results and Discussion

### 3.1. Molecular Identification

Amplification of fragments of the *β*-giardin gene and the 220-bp fragment of the GDH gene demonstrated the presence of* G. duodenalis* in the fecal samples from the University of Campinas (UNICAMP) Clinical Hospital.

Although* G. duodenalis* infects humans, a wide range of species can be found in many other mammals [27]. Based on genetic analysis,* G. duodenalis* can be considered a complex species, whose members exhibit little variation in their morphology (designated assemblages A to G). The sequencing reaction that was performed for the amplified fragment from the GDH gene, using DNA from a positive sample in this study, confirmed the presence of* G. duodenalis* assemblage A.

Assemblages A and B are broadly distributed responsible for human infection and are found in a range of mammals; however, the role of animals in the epidemiology of human infection remains unclear, despite the fact that the zoonotic potential of* Giardia* spp. was recognized by the World Health Organization (WHO) 30 years ago [3].

### 3.2. Occurrence of* Giardia* spp. Cysts in Atibaia River Samples

*Giardia* spp. cysts were detected, with 97.5% of surface water samples with average 7,9 × 10^5^ cysts L^−1^. Only one sample resulted negative for cysts detection.* Giardia* spp. are constantly found in Atibaia River; this is because the load of fecal material discharged in this river through domestic effluents is significant [12].

### 3.3. Reduction in the Number of* G. duodenalis* Cysts

**Figure 3:**
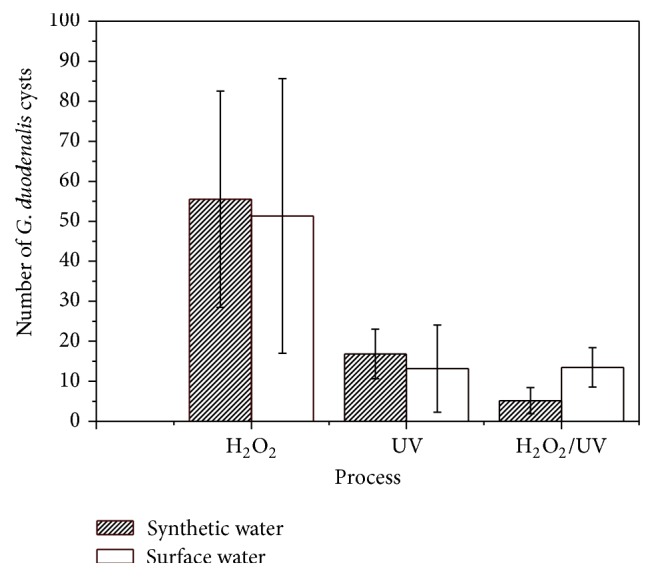
Average number (and standard deviation) of* G. duodenalis* cysts recovered from synthetic and surface water.

A reduction in the number of organisms that were initially inoculated, in relation to the number of organisms recovered after the water treatment process is applied, may be considered a measure of the efficiency of the processes [36, 37]. An average of 2.5 × 10^3^ *G. duodenalis* cysts were inoculated in all experiments. According to the results obtained in this study, AOP treatment was responsible for a greater reduction in* G. duodenalis* cysts in comparison to chemical or physical processes evaluated alone (Figure 3).

As shown in Table 1, AOP resulted in the highest reduction in* G. duodenalis* cysts counted in every condition tested.

However, for surface water, AOP and UV showed higher efficiency in reducing the number of organisms only when compared with peroxidation (Table 1). When comparing photolysis to AOP, the results presented no statistically significant difference (AOP = UV > H_2_O_2_).

Efficiencies for every process were calculated considering inoculum and reductions in cysts count (Table 1).

AOP is capable of degrading organic compounds by promoting chemical modifications of the substrate, which enables their complete mineralization in some cases [38]. Thus, due to the nonselective character of the hydroxyl radical, the organic matter present in water most likely sequesters part of these radicals that were generated by the AOP, which causes a reduction of its action on the* G. duodenalis* cyst walls.

Three main effects of particulate matter can impact the ability of UV radiation to inactivate* G. duodenalis*: (a) the number and size of particles, which could cause the dispersion of radiation and the shading of certain areas; (b) the nature of the particles (whether organic or inorganic); and (c) the degree of association between the parasitic stage and the particles, which exerts a protective effect on the pathogenic organisms [38–40].

The surface water samples turbidity was from 22 to 34 NTU, with a mean value of 27 NTU. The interference of the particulate matter with the efficiency of UV radiation is complex; all previously described issues can explain the higher efficiency of the AOP when driven in synthetic water and the decrease in efficiency when applied to surface water.

The efficiency in the reduction of cysts in synthetic and surface water achieved by photolysis presented no statistically significant difference.

A wide range of UV doses is reported in related research: 1 mJ cm^−2^ to 40,000 mJ cm^−2^ is considered effective in inactivating protozoa [30, 41]; thereby, the evaluation of different doses of radiation is relevant.

Based on research related to protozoan inactivation and considering the high resistance of protozoa to disinfection, a high concentration of hydrogen peroxide was chosen [28, 29, 42]. However, this concentration may have underestimated the efficiency of the AOP in reducing the number of* G. duodenalis* cysts present in surface water. When excessive H_2_O_2_ is present, it may become a significant competitor for the ^•^OH radicals and a generator of hydroperoxide radicals (HO_2_^•^), which have less reactive potential than the ^•^OH radicals. These reactions consume the hydroxyl radical, which decreases the oxidation potential of the medium [43]. An optimum concentration of hydrogen peroxide is recommended to improve process efficiency.

### 3.4. Fluorescence Reduction in* G. duodenalis* Cysts

In synthetic water, 35,5%, 23,4%, and 55,4% of the cysts exhibited weak fluorescence when using the AOP, peroxidation, and photolysis processes, respectively, suggesting that the sequence of process efficiency damaging the wall of the protozoa is UV > AOP = H_2_O_2_ (no statistically significant difference between AOP and H_2_O_2_ process was observed).

When surface water was evaluated, AOP was more effective in decreasing the fluorescence of the wall surfaces of* G. duodenalis* cysts. By using the AOP, peroxidation, and photolysis processes, 98,7%, 62,6%, and 79,7%, respectively, of the* G. duodenalis* cysts presented weak fluorescence (AOP > UV = H_2_O_2_).

During the disinfection process, the destruction of the microorganisms is directly related to disinfectant concentration and time of exposure of the cells to the disinfectant. This damage is caused by reactions that involve intracellular and extracellular biomolecules. Inactivation will occur when vital constituents suffer a certain level of irreversible damage [44].

**Figure 4:**
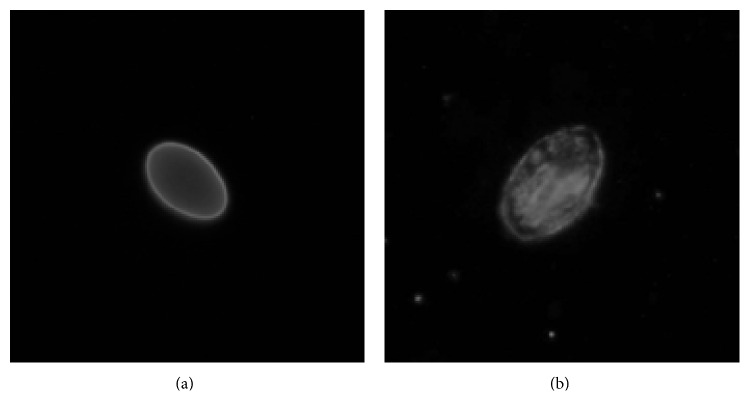
*G. duodenalis* cysts: (a) typical fluorescence (400x) and (b) weak fluorescence (600x).

The ability of hydroxyl radicals to destroy many types of macromolecules, including carbohydrates, nucleic acids, lipids, and amino acids, is very strong. Previous studies of the filamentous walls of* Giardia* spp. cysts have confirmed the presence of materials such as carbohydrates and protein in a ratio of 3 : 2 (w/w) [45]. The decrease of fluorescence of cysts is an evidence of serious damage to the cysts wall due to hydroxyl radicals (Figures 4(a) and 4(b)).

França [46] also observed a reduction in the number of organisms and a decrease in the fluorescence of* G. duodenalis* cysts that were submitted to photoelectrochemical treatment for 90 minutes.

The AOP (H_2_O_2_/UV) using lower concentration of hydrogen peroxide (15 and 21 mg L^−1^) and lower dose of UV radiation (44 mJ cm^−2^) has shown itself as a technology capable of causing damage in* Cryptosporidium* spp. oocysts and* G. duodenalis* cysts [47].

Evaluation of the efficiency of the disinfection processes in the environmental samples is a difficult task due to the presence of interfering material dispersed in the medium. AOP was more efficient than peroxidation and photolysis in reducing the number and/or fluorescence of the* G. duodenalis* cyst wall, suggesting protozoan inactivation. Studies with low concentrations of hydrogen peroxide and a low dose of UV AOP to increase the efficiency of hydroxyl radicals are needed to elucidate the AOP action in resistant forms of protozoa.

## 4. Conclusion

AOP (H_2_O_2_/UV) was effective in reducing the number of cysts of* G. duodenalis* when they were inoculated in synthetic water and in damaging the wall of cysts when present in surface water with 86,2% and 98,7%, respectively.

## Figures and Tables

**Table 1:** Efficiency (%) of the three processes in reducing the number of *G.  duodenalis* cysts, for each water type evaluated.

Matrix	Processes		
	H_2_O_2_	UV	H_2_O_2_/UV
Synthetic water	0	55,1	86,2
Surface water	0	64,9	64,0

## References

[1] Moe C. L., Rheingans R. D. (2006). Global challenges in water, sanitation and health. *Journal of Water and Health*.

[2] Baldursson S., Karanis P. (2011). Waterborne transmission of protozoan parasites: Review of worldwide outbreaks—an update 2004–2010. *Water Research*.

[3] Sprong H., Cacciò S. M., van der Giessen J. W., ZOOPNET network and partners (2009). Identification of zoonotic genotypes of Giardia duodenalis. *PLoS Neglected Tropical Diseases*.

[4] Machado M., Dinis A. M., Salgueiro L., Custódio J. B. A., Cavaleiro C., Sousa M. C. (2011). Anti-*Giardia* activity of *Syzygium aromaticum* essential oil and eugenol: effects on growth, viability, adherence and ultrastructure. *Experimental Parasitology*.

[5] Cantey P. T., Roy S., Lee B. (2011). Study of nonoutbreak giardiasis: novel findings and implications for research. *The American Journal of Medicine*.

[6] Neto R. C., Santos J. U., Franco R. M. B. (2006). Evaluation of activated sludge treatment and the efficiency of the disinfection of *Giardia* species cysts and *Cryptosporidium* oocysts by UV at a sludge treatment plant in Campinas, South-East Brazil. *Water Science and Technology*.

[7] Nasser A. M., Vaizel-Ohayon D., Aharoni A., Revhun M. (2012). Prevalence and fate of giardia cysts in wastewater treatment plants. *Journal of Applied Microbiology*.

[8] Robertson L. J., Hermansen L., Gjerde B. K., Strand E., Alvsvåg J. O., Langeland N. (2006). Application of genotyping during an extensive outbreak of waterborne giardiasis in Bergen, Norway, during autumn and winter 2004. *Applied and Environmental Microbiology*.

[9] Thompson R. C. A., Monis P. (2012). Giardia—from genome to proteome. *Advances in Parasitology*.

[10] Ministério das Cidades (MC)-Sistema Nacional de Informação sobre Saneamento (SNIS)-Diagnóstico dos Serviços de Água e Esgoto-2011.

[11] Araújo R. S., Dropa M., Fernandes L. N. (2011). Genotypic characterization of cryptosporidium hominis from water samples in São Paulo, Brazil. *The American Journal of Tropical Medicine and Hygiene*.

[12] Neto R. C., dos Santos L. U., Sato M. I. Z., Franco R. M. B. (2010). *Cryptosporidium* spp. and *Giardia* spp. in surface water supply of Campinas, Southeast Brazil. *Water Science & Technology*.

[13] Leal Diego A. G., Dores Ramos A. P., Marques Souza D. S. (2013). Sanitary quality of edible bivalve mollusks in Southeastern Brazil using an UV based depuration system. *Ocean and Coastal Management*.

[14] Santos L. U., Bonatti T. R., Cantusio Neto R., Franco R. M. B. (2004). Occurrence of *Giardia* cysts and *Cryptosporidium* oocysts in activated sludge samples in Campinas, SP, Brazil. *Revista do Instituto de Medicina Tropical de Sao Paulo*.

[15] Hayes J. C., Garvey M., Fogarty A. M., Clifford E., Rowan N. J. (2012). Inactivation of recalcitrant protozoan oocysts and bacterial endospores in drinking water using high-intensity pulsed UV light irradiation. *Water Science and Technology: Water Supply*.

[16] Mun S., Cho S. H., Kim T. S., Oh B. T., Yoon J. (2009). Inactivation of Ascaris eggs in soil by microwave treatment compared to UV and ozone treatment. *Chemosphere*.

[17] Hoigne J., Bader H. (1976). The role of hydroxyl radical reactions in ozonation processes in aqueous solutions. *Water Research*.

[18] Sievers M. (2011). Advanced oxidation processes. *Treatise on Water Science*.

[19] da Silva C. R., Maniero M. G., Rath S., Guimarães J. R. (2011). Antibacterial activity inhibition after the degradation of flumequine by UV/H_2_O_2_. *Journal of Advanced Oxidation Technologies*.

[20] Guimarães J. R., Barretto A. S. (2003). Photocatalytic inactivation of *Clostridium perfringens* and coliphages in water. *Brazilian Journal of Chemical Engineering*.

[21] Rodrigues C. P., Ziolli R. L., Guimarães J. R. (2007). Inactivation of *Escherichia coli* in water by TiO_2_-assisted disinfection using solar light. *Journal of the Brazilian Chemical Society*.

[22] Guimarães J. R., Ibáñez J., Litter M. I., Pizarro R. (2004). *Eliminación de contaminantes por fotocatálisis heterogénea*.

[23] Curtis T. P., Alker G. W., Dowling B. M., Christensen P. A. (2002). Fate of *Cryptosporidium oocysts* in an immobilised titanium dioxide reactor with electric field enhancement. *Water Research*.

[24] Ryu H., Gerrity D., Crittenden J. C., Abbaszadegan M. (2008). Photocatalytic inactivation of *Cryptosporidium parvum* with TiO_2_ and low-pressure ultraviolet irradiation. *Water Research*.

[25] APHA-AWWA-WEF (1998). *Standard Methods for the Examination of Water and Wastewater*.

[26] Smith A. L., Smith H. V. (1989). A comparison of fluorescein diacetate and propidium iodide staining and *in vitro* excystation for determining *Giardia intestinalis* cyst viability. *Parasitology*.

[27] Barbee S. L., Weber D. J., Sobsey M. D., Rutala W. A. (1999). Inactivation of *Cryptosporidium parvum* oocyst infectivity by disinfection and sterilization processes. *Gastrointestinal Endoscopy*.

[28] Hernández F., Hernández D., Zamora Z. (2009). *Giardia duodenalis*: effects of an ozonized sunflower oil product (Oleozon) on *in vitro* trophozoites. *Experimental Parasitology*.

[29] Weir S. C., Pokorny N. J., Carreno R. A., Trevors J. T., Lee H. (2002). Efficacy of common laboratory disinfectants on the infectivity of *Cryptosporidium parvum* oocysts in cell culture. *Applied and Environmental Microbiology*.

[30] Clancy J. L., Bukhari Z., Hargy T. M., Bolton J. R., Dussert B. W., Marshall M. M. (2000). Using UV to inactivate *Cryptosporidium*. *American Water Works Association*.

[31] Gómez-Couso H., Fontán-Sainz M., McGuigan K. G., Ares-Mazás E. (2009). Effect of the radiation intensity, water turbidity and exposure time on the survival of *Cryptosporidium* during simulated solar disinfection of drinking water. *Acta Tropica*.

[32] Franco R. M. B., Rocha-Eberhardt R., Cantusio Neto R. (2001). Occurrence of *Cryptosporidium oocysts* and *Giardia* cysts in raw water from the Atibaia river, Campinas, Brazil. *Revista do Instituto de Medicina Tropical de Sao Paulo*.

[33] Mahbubani M. H., Bej A. K., Perlin M. H., Schaefer F. W., Jakubowski W., Atlas R. M. (1992). Differentiation of *Giardia duodenalis* from other *Giardia* spp. by using polymerase chain reaction and gene probes. *Journal of Clinical Microbiology*.

[34] Abe N., Kimata I., Iseki M. (2003). Identification of genotypes of *Giardia intestinalis* isolates from dogs in Japan by direct sequencing of the PCR amplified glutamate dehydrogenase gene. *Journal of Veterinary Medical Science*.

[35] U.S. Environmental Protection Agency (EPA) (2012). *Method 1623.1: Cryptosporidium and Giardia in Water by Filtration/IMS/FA*.

[36] Hijnen W. A. M., Beerendonk E. F., Medema G. J. (2006). Inactivation credit of UV radiation for viruses, bacteria and protozoan (oo)cysts in water: a review. *Water Research*.

[37] Otaki M., Hirata T., Ohgaki S. (2000). Aqueous microorganisms inactivation by photocatalytic reaction. *Water Science and Technology*.

[38] Doménech X., Jardim W. F., Litter M. I., Doménech X., Jardim W. F., Litter M. I. (2001). Procesos avanzados de oxidación para la eliminación de contaminantes. *Eliminación de Contaminantes por Fotocatálisis Heterogenea, La Plata*.

[39] Caron E., Chevrefils G., Barbeau B., Payment P., Prevost M. (2007). Impact of microparticles on UV disinfection of indigenous aerobic spores. *Water Research*.

[40] Vogelpohl A. (2007). Applications of AOPs in wastewater treatment. *Water Science and Technology*.

[41] Shin G.-A., Linden K. G., Faubert G. (2009). Inactivation of *Giardia lamblia* cysts by polychromatic UV. *Letters in Applied Microbiology*.

[42] Quilez J., Sanchez-Acedo C., Avendaño C., del Cacho E., Lopez-Bernad F. (2005). Efficacy of two peroxygen-based disinfectants for inactivation of *Cryptosporidium parvum* oocysts. *Applied and Environmental Microbiology*.

[43] Hu Q., Zhang C., Wang Z. (2008). Photodegradation of methyl tert-butyl ether (MTBE) by UV/H_2_O_2_ and UV/TiO_2_. *Journal of Hazardous Materials*.

[44] Fernando W. J. (2009). Theoretical considerations and modeling of chemical inactivation of microorganisms: inactivation of *Giardia Cysts* by free chlorine. *Journal of Theoretical Biology*.

[45] Sökmen M., Degerli S., Aslan A. (2008). Photocatalytic disinfection of *Giardia intestinalis* and *Acanthamoeba castellani cysts* in water. *Experimental Parasitology*.

[46] França R. B. (2007). *Cryptosporidium spp., Giardia spp. e ovos de helmintos em esgoto hospitalar: destruição e análise do dano estrutural dos protozoários após o processo fotoeletroquímico, Dissertação (Mestrado em Parasitologia) [M.S. thesis]*.

[47] Guimarães J. R., Santos L. U., Franco R. M. B., Guadagnini R. A. Peroxidation and peroxidation assisted by ultraviolet radiation in the inactivation of Giardia duodenalis (Peroxidação e peroxidação assistida por radiação ultravioleta na inativação de cistos de *Giardia duodenalis*).

